# The role of resting-state functional MRI for clinical preoperative language mapping

**DOI:** 10.1186/s40644-020-00327-w

**Published:** 2020-07-11

**Authors:** Vinodh A. Kumar, Islam M. Heiba, Sujit S. Prabhu, Melissa M. Chen, Rivka R. Colen, Angela L. Young, Jason M. Johnson, Ping Hou, Kyle Noll, Sherise D. Ferguson, Ganesh Rao, Frederick F. Lang, Donald F. Schomer, Ho-Ling Liu

**Affiliations:** 1 Department of Neuroradiology, The University of Texas MD Anderson Cancer Center, Houston, TX USA; 2 Department of Neurosurgery, The University of Texas MD Anderson Cancer Center, Houston, TX USA; 3 Department of Diagnostic Radiology, The University of Pittsburgh Medical Center, Pittsburgh, PA USA; 4 Department of Imaging Physics, The University of Texas MD Anderson Cancer Center, Houston, TX USA; 5 Department of Neuro-Oncology, The University of Texas MD Anderson Cancer Center, Houston, TX USA

**Keywords:** Functional connectivity, Resting-state fMRI, Seed-based correlation, Regional homogeneity

## Abstract

**Background:**

Task-based functional MRI (tb-fMRI) is a well-established technique used to identify eloquent cortex, but has limitations, particularly in cognitively impaired patients who cannot perform language paradigms. Resting-state functional MRI (rs-fMRI) is a potential alternative modality for presurgical mapping of language networks that does not require task performance. The purpose of our study is to determine the utility of rs-fMRI for clinical preoperative language mapping when tb-fMRI is limited.

**Methods:**

We retrospectively reviewed 134 brain tumor patients who underwent preoperative fMRI language mapping. rs-fMRI was post-processed with seed-based correlation (SBC) analysis, when language tb-fMRI was limited. Two neuroradiologists reviewed both the tb-fMRI and rs-fMRI results. Six neurosurgeons retrospectively rated the usefulness of rs-fMRI for language mapping in their patients.

**Results:**

Of the 134 patients, 49 cases had limited tb-fMRI and rs-fMRI was post-processed. Two neuroradiologists found rs-fMRI beneficial for functional language mapping in 41(84%) and 43 (88%) cases respectively; Cohen’s kappa is 0.83, with a 95% confidence interval (0.61, 1.00). The neurosurgeons found rs-fMRI “definitely” useful in 26 cases (60%) and “somewhat” useful in 13 cases (30%) in locating potential eloquent language centers of clinical interest. Six unsuccessful rs-fMRI cases were due to: head motion (2 cases), nonspecific functionality connectivity outside the posterior language network (1 case), and an unknown system instability (3 cases).

**Conclusions:**

This study is a proof of concept that shows SBC rs-fMRI may be a viable alternative for clinical language mapping when tb-fMRI is limited.

## Background

Functional MRI (fMRI) is a well-known modality often used in neurosurgical oncology for preoperative planning to facilitate safe, maximal surgical resection of tumors located in eloquent brain areas, including those in language areas [1, 2]. Language mapping with task-based fMRI (tb-fMRI) requires a combination of multiple language paradigms to generate reliable and accurate activation of language networks [1]. tb-fMRI also requires highly trained personnel to determine patient cognition and to choose suitable language paradigms. Thus, tb-fMRI can be challenging in brain tumor patients who may be cognitively impaired and are unable to perform required tasks.

Resting-state fMRI (rs-fMRI) is an alternative modality for preoperative mapping of language and motor networks and has the potential to overcome some limitations of tb-fMRI [3]. rs-fMRI does not depend on patient performance, but rather on the detection of spontaneous changes in low-frequency blood oxygenation level-dependent (BOLD) oscillations to identify associated brain areas while the patient is at rest [4]. Further, rs-fMRI can be successfully performed when the patient is asleep or even under anesthesia [5]. Tie et al. showed in healthy right-handed individuals, that language networks obtained from rs-fMRI revealed highly similar overlap with language networks obtained from tb-fMRI, especially in the left frontal and temporal/parietal regions [6]. These results were also reproduced in patients with brain tumors and epilepsy [7]. In addition, Rosazza et al. [8] reported a significant correlation of the results of rs-fMRI seed-based correlation (SBC) and independent component analysis (ICA) in 40 healthy individuals.

To the best of our knowledge, no previous studies have examined the use of SBC rs-fMRI for clinical language mapping in a large series of brain tumor patients*.* The purpose of our study is to determine the utility of SBC rs-fMRI for preoperative language mapping when tb-fMRI is limited.

## Methods

### Subjects

Our institutional review board approved this retrospective study (PA19–0417). A total of 134 patients with brain tumors underwent both tb-fMRI and rs-fMRI for preoperative language mapping between September 1, 2017 and March 31, 2019. Eighty-five patients underwent successful tb-fMRI language mapping, and, thus, rs-fMRI post-processing was not performed. In the remaining 49 patients, rs-fMRI was processed; in the latter, either the patient could not perform the tb-fMRI paradigms, the patient performed the tb-fMRI paradigms poorly, the results of the tb-fMRI activation specific to the language area near tumor was equivocal or there was excessive patient motion. In these cases, tb-fMRI was defined as “limited”. We collected the clinical and fMRI data for each of these 49 patients.

### Imaging techniques

#### MRI acquisition

Structural MRI and fMRI were performed using a 3 T GE MR750 scanner (GE Healthcare, Waukesha, Wisconsin) with an 8-channel head coil. Functional images were acquired using a T2*-weighted gradient-echo echo-planar imaging sequence (repetition time/echo time = 2000/25 ms, matrix size = 64 × 64, field of view = 24 × 24 cm, 32 slices, slice thickness = 4 mm with no gap). Thirty-two slices were acquired per dynamic to cover the entire brain. High-resolution T_2_-weighted FLAIR and 3D spoiled gradient-echo T1-weighted sequences were acquired for anatomic reference. A six-minute rs-fMRI acquisition was obtained prior to language tb-fMRI in all cases. During this acquisition, the patient was asked to do the following: close their eyes, not fall asleep, clear their mind, and keep their head still.

#### Tb-fMRI paradigms

The following pertains to the 49 cases in which the tb-fMRI results were limited. In 46 English speaking patients, three fMRI language paradigms were administered in English: letter fluency, category fluency, and sentence completion. There were three patients who did not speak English. The category fluency and object naming paradigms were administered in Mandarin in one patient and in Arabic in another patient. The object naming paradigm alone was administered in one Arabic speaking patient.

All paradigms used alternating active and control blocks. Before tb-fMRI, all patients underwent practice trials according to our standard preoperative mapping guidelines to ensure that the patient could perform the tasks correctly. During fMRI acquisition, each paradigm was displayed using an MRI-compatible 32-in. liquid crystal display, and oral instructions were provided through an intercom.

#### Workflow and processing pipeline (tb-fMRI & rs-fMRI)

Task-based (tb) fMRI data were processed by using the DynaSuite Neuro software, version 3.0 (Invivo, Philips, Gainesville, Florida). Image pre-processing for fMRI included motion correction and spatial smoothing with a 4-mm full width at half-maximum Gaussian kernel. A functional activation map was created using the correlation analysis of the task paradigms convolved with a canonical hemodynamic response function and the signal intensity time course for each voxel. Statistical thresholds ranging from corrected *p* < 10^− 6^ to 10^− 2^ were applied to optimize the visualization of language areas.

Resting-state fMRI data were processed by using an in-house software, IClinfMRI [9], under MATLAB 2014a (The MathWorks, Inc., Natick, Massachusetts). The software calls functions in free for non-commercial use software including dcm2nii (https://www.nitrc.org/projects/dcm2nii/), AFNI (version 16.2.09) [10] and SPM12 (v6685) (Welcome Department of Cognitive Neurology, Institute of Neurology, London, UK). The resting-state datasets were pre-processed through slice timing, motion correction, de-spiking, detrending, regressing out covariates (including six motion parameters and two averaged fluctuations over masks of white matter and cerebrospinal fluid), band-pass filtering of 0.01–0.08 Hz, and 4-mm FWHM smoothing. After the pre-processing, seed-based analysis was applied to detect the language network.

Two approaches were used for the seed selection: (1) by referencing to tb-fMRI activations (when available) or (2) by referencing a regional homogeneity (ReHo) map [11]. When using the first approach, peak tb-fMRI activations in a primary language area apart from tumor were used as seed locations. For example, when the tumor was close to the anterior language area, the seed was selected in the posterior language area, and vice versa. When using the second approach, peaks of the ReHo map within an anatomical mask of the anterior or posterior language network apart from tumor were used as seed locations. The anatomical mask was generated based on the meta-analysis result obtained from Neurosynth (http://neurosynth.org/) after the term “language” was entered [12]. The mask was constrained within the posterior inferior frontal gyrus, Broca’s area (pars triangularis and par opercularis), and middle frontal gyrus for the anterior language area (ALA) and within angular gyrus, supramarginal gyrus, and superior and middle temporal gyri for the posterior language area (PLA) [13]. The LONI Probabilistic Brain Atlas was used to apply the anatomical constraints [14]. When the ReHo map was used to help seed localization, we would attempt seeding at multiple local maxima within the anatomical areas outlined by the meta-analysis mask. The final FC map was qualitatively determined by (1) the existence of positive functional connectivity (FC) to the ALA or PLA of clinical interest and (2) minimizing non-specific FC such as in the CSF spaces. In addition, the presence of FC in secondary language areas (i.e. language supplementary motor area and the visual word form area) increased diagnostic confidence in the final FC map. This manual searching process took on average 10 attempts for each case from which the final rs-fMRI FC map was selected.

Details in the implementation of the seed selection methods are described in Hsu et al. [9]. As part of our clinical SBC rs-fMRI post-processing workflow at the time of this study, the tb-fMRI activation approach was attempted first. If tb-fMRI activation was not available or if this method of seeding was unsuccessful, then the regional homogeneity method was utilized. For each seed location, a sphere of 6-mm radius was defined as the seed region and a reference time course was generated by averaging the time courses over the voxels within the region. The rs-fMRI connectivity map was computed using Pearson correlation between the reference time course and that of each voxel in the brain (voxel size = 3.75 × 3.75 × 4 mm^3^). The correlation coefficient map was then converted to a Fisher’s z map. A Z-value threshold ranged from 0.6 to 1.0 was applied to optimize the visualization of the language network. For each patient, multiple seeds were selected and each generated a functional connectivity map. The final results were determined by one of two clinical imaging physicists (P.H. and H-L.L.; both with more than 10 years of clinical fMRI experience) who processed the rs-fMRI data. Of note, > 1 mm translation or > 1 degree rotation in any direction for all post processed tb-fMRI and rs-fMRI cases were regarded as motion degraded.

### Neuroradiology assessment

Two neuroradiologists (V.A.K. and M.C.), with expertise in clinical fMRI, retrospectively reviewed the fMRIs from patients whose tb-fMRI data were deemed limited and rs-fMRI data were subsequently post-processed. In these limited tb-fMRI cases (1) the patient was too impaired to perform tb-fMRI, (2) the patient performed poorly during tb-fMRI per clinician observation during scanning, (3) had no to weak tb-fMRI BOLD signal specific to the language area of clinical interest near the tumor, (4) had nonspecific BOLD activation or (5) there was significant patient motion during tb-fMRI acquisition as determined by motion graphs. Nonspecific activation is defined as BOLD activation outside the previously defined anterior or posterior language areas.

To evaluate the benefit of SBC rs-fMRI for preoperative functional language mapping, the neuroradiologists first *independently* reviewed the post-processed rs-fMRI data. A “yes” was recorded if the generated FC was within the previously defined ALA or PLA of clinical interest near tumor. A “no” was recorded, if the FC was outside the defined ALA or PLA of clinical interest, if any rs-fMRI artifacts were observed, or for any other reason the neuroradiologist was not confident in the rs-fMRI results. If there was a discrepancy in agreement between the 2 neuroradiologists; these cases were then reviewed together and a consensus “yes” or “no” was recorded.

### Neurosurgery assessment

The six referring neurosurgeons were provided a questionnaire to assess the clinical value of rs-fMRI in the preoperative mapping of language centers when tb-fMRI proved insufficient. The questionnaire included the following question: When tb-fMRI was limited, *based on your knowledge of neuroanatomical functional areas,* did you find the rs-fMRI data “useful” in locating a potential eloquent language area near tumor to assist direct cortical stimulation in this case? Neurosurgeon rating for each case: 1) not useful; 2) neutral; 3) somewhat useful; 4) definitely useful; or 5) not applicable.” The questionnaire responses were collected and recorded.

### Statistical analysis

The agreement between the two neuroradiologists’ rating of the benefit of the post-processed rs-fMRI data for presurgical language mapping was assessed using Cohen’s kappa statistic.

## Results

Patient demographics for whom resting-state fMRI was post-processed are summarized in Table 1.

**Table 1:** Patient demographics for whom resting-state functional MRI was performed (*n* = 49)

Characteristic	No. of patients
Mean age (range)	47.5 years (17–78 years)
Sex	
Male	28
Female	21
Hand dominance	
Right	45
Mixed	2
Left	2
Tumor location	
Right hemisphere	1
Frontotemporal	1
Left hemisphere	48
Frontal	17
Parietal	7
Temporal	18
Insular	1
Frontoparietal	2
Temporoparietal	2
Intraventricular	1
Tumor enhancement	
Enhancing	39
Non-enhancing	10
Surgery	
Awake	37
Asleep	11
Canceled	1
Pathology	
Grade II astrocytoma (WT:5; MUT:4; NOS:1)	10
Anaplastic astrocytoma (WT:2; MUT: 3)	5
Glioblastoma (WT:13; MUT: 8, NOS:1)	22
Oligodendroglioma	6
Metastasis	3
Pleomorphic xanthoastrocytoma	1
Anaplastic pleomorphic xanthoastrocytoma	2

*WT* Wild type, *MUT* Mutant, *NOS* Not otherwise specified

### Causes of limited tb-fMRI

Of the 134 patients with brain tumors who were evaluated for preoperative fMRI language mapping, 85 had successful tb-fMRI results and 49 had limited tb-fMRI results. Thus, for these 49 patients, rs-fMRI was post-processed. The following lists the causes of no or limited tb-fMRI data: 1) poor patient performance as determined by the neuropsychologist during the fMRI study (*n* = 12, 24.5%); 2) neuropsychological testing before the fMRI revealed that the patients were too impaired (e.g., aphasic) to attempt tb-fMRI (*n* = 7, 14.3%); 3) weak BOLD activation in language area near tumor (*N* = 9; 18.4%); 4) no BOLD activation near tumor (*N* = 8, 16.3%); 5) nonspecific BOLD activation outside the previously defined ALA or PLA (*n* = 11; 22.4%); 6) patient motion artifact (*n* = 2; 4.1%). There were no tb-fMRI technical (hardware or software) failures and no tb-fMRI failures due to susceptibility artifacts.

### Resting-state fMRI seed placement

For the 49 cases, the rs-fMRI seed location and resultant functional connectivity to the language area of clinical interest is summarized in Table 2.

**Table 2:** Resting-state functional MRI findings (*n* = 49 patients)

	No. of patients
Method of resting-state seed placement	
Task-based BOLD activation	33
Regional homogeneity maps	16
Resting-state seed location **➔** functional connectivity location	
Left ALA **➔** Left PLA	20
Left PLA **➔** Left ALA	19
Right PLA **➔** Right ALA	1
Right ALA **➔** Left ALA	2
Right ALA **➔** Left PLA	1
Resting-state functional MRI failure	
Patient head motion	2
Nonspecific location of functional connectivity	1
Unknown system instability	3

*ALA* anterior langauge area, *PLA* posterior language area

### Neuroradiological assessment

Two neuroradiologists independently reviewed rs-fMRI data from the 49 patients in which tb-fMRI was limited. One neuroradiologist determined that the post-processed rs-fMRI data was beneficial for language mapping in 41 patients and the other determined that it was beneficial in 43 patients. Cohen’s kappa is 0.83, with a 95% confidence interval (0.61, 1.00). With regard to the 2 patients in whom there was a discrepancy in agreement, the first neuroradiologist felt that tb-fMRI suggested language reorganized to the contralateral ALA in two patients. The other neuroradiologist felt that the rs-fMRI results were still of value in these two patients in the event that reorganization was incomplete and residual language function remained in the ALA area ipsilateral to the tumor. After consensus review, both neuroradiologists deemed these 2 rs-fMRI cases helpful and therefore a total of 43 cases were found beneficial for language mapping.

The neuroradiologists independently deemed that the rs-fMRI data was not diagnostic and did not benefit preoperative language mapping in 6 cases (12%). In two cases, the unsuccessful rs-fMRI was attributed to patient head motion during the rs-fMRI sequence acquisition. This was determined by reviewing the rs-fMRI motion graphs. In one patient, the FC obtained was in a nonspecific anatomic location outside the previously defined PLA. The remaining 3 cases of rs-fMRI failure were due to an unknown system instability.

### Neurosurgical assessment

The six cases deemed rs-fMRI failures by both neuroradiologists were not included for neurosurgery assessment. The neuroradiologists submitted the remaining 43 rs-fMRI FC results for neurosurgery assessment. The six neurosurgeons each reviewed the rs-fMRI FC results for their own patients. The neurosurgeons found the post-processed rs-fMRI results to be “definitely” useful in 26 patients (60%) and “somewhat” useful in 13 patients (30%) for localizing potential anterior and posterior language areas and thus providing potential stimulation points near tumor to assist direct cortical stimulation. For two patients (5%), a neutral rating was rendered. A “not applicable” rating was given for two patients (5%). In the first, the surgery was canceled. In the other, rs-fMRI showed good ALA FC, but this was not of interest to the surgeon because he had no plan to resect near this area. There were no negative ratings of the rs-fMRI data by the neurosurgeons.

### Illustrative cases

#### Case 1: successful rs-fMRI after no tb-fMRI activation

**Fig. 1:**
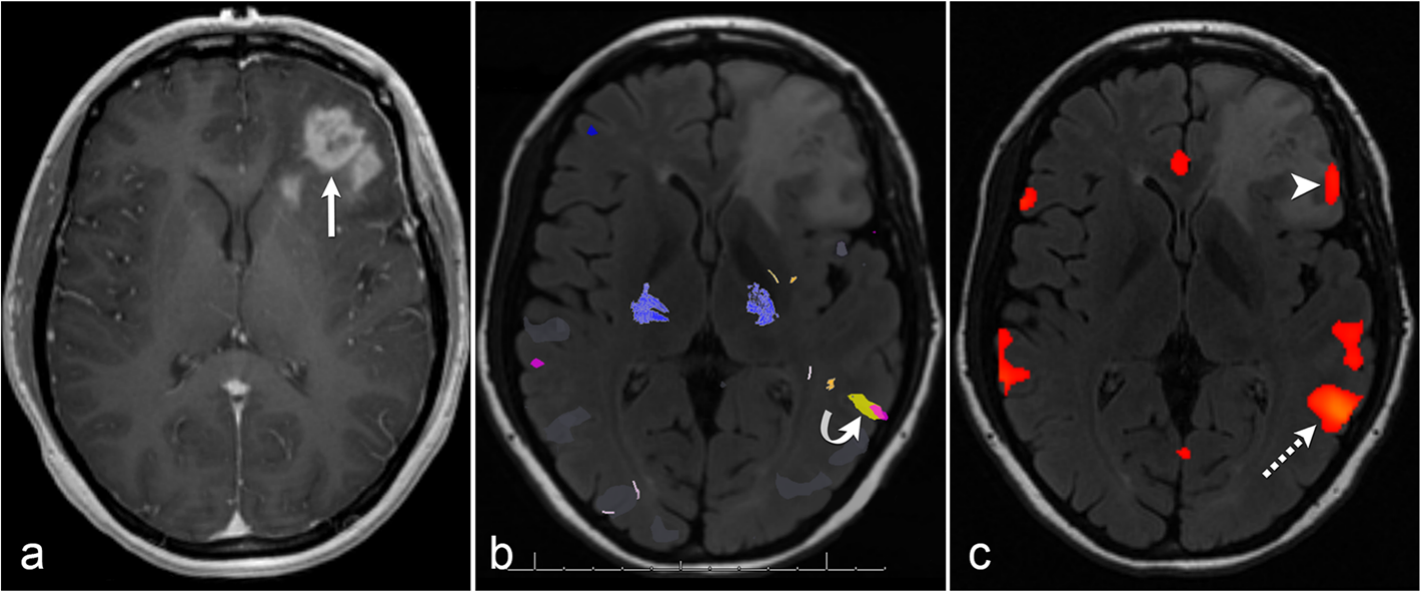
Successful resting-state fMRI after no task-based fMRI activation. Post-contrast axial T1 image (**a**) shows a left frontal glioblastoma (arrow). Task-based fMRI (**b**) demonstrates no BOLD activation in left anterior language area and robust BOLD activation related to the sentence completion paradigm (yellow) and category fluency paradigm (pink) in left Wernicke’s area (curved arrow). Resting-state fMRI (**c**) with seed placement in Wernicke’s area at site of task-based BOLD activation (dashed arrow) results in robust functional connectivity to left Broca’s area (arrowhead)

In a 24-year-old right-handed woman with an *IDH* mutant recurrent left frontal glioblastoma, tb-fMRI was performed but showed no BOLD activation in the left anterior language area; therefore, rs-fMRI was post-processed. The tb-fMRI activation in left Wernicke’s area was used as a seed for rs-fMRI. Functional connectivity was successfully found in left Broca’s area (Fig. 1).

#### Case 2: successful rs-fMRI after limited tb-fMRI due to patient motion

**Fig. 2:**
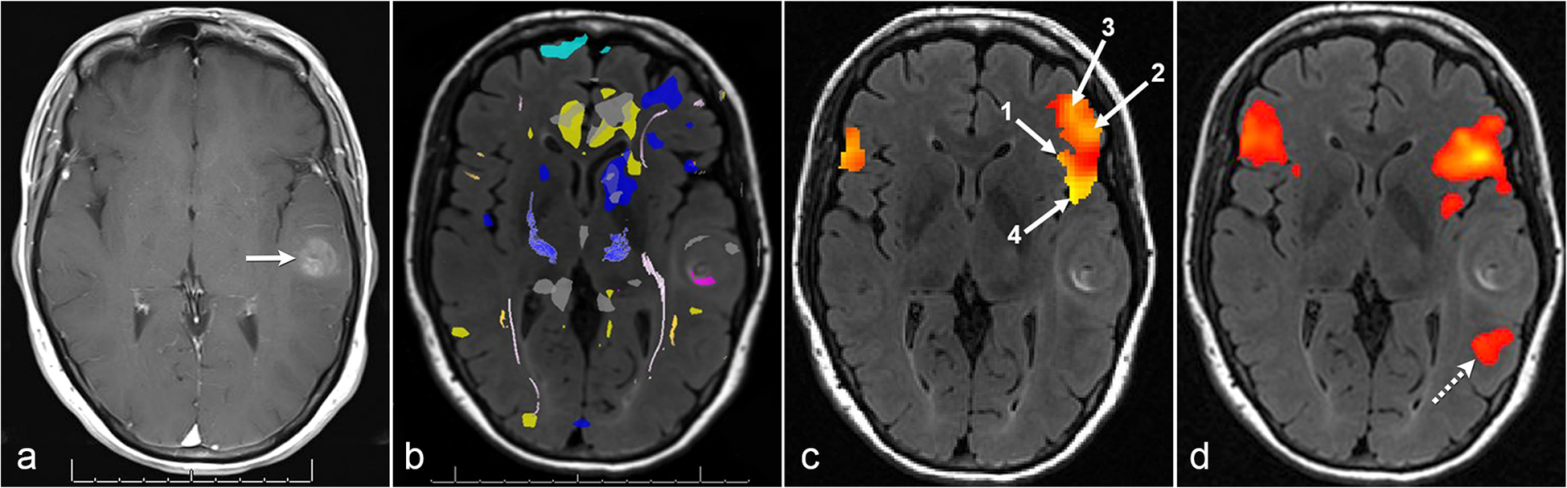
Successful resting-state fMRI after patient motion limited task-based fMRI. Post-contrast axial T1 image (**a**) shows a left temporal glioblastoma (arrow). Task-based fMRI (**b**) displays nonspecific BOLD activations (i.e. yellow = sentence completion; blue = letter fluency) and noise due to patient motion. This case illustrates a regional homogeneity functional connectivity map confined to the Broca’s area meta-analysis (**c**) showing 4 seed candidates. Seed #1 results in the final resting-state fMRI functional connectivity map (**d**) which demonstrates robust connectivity in left Wernicke’s area (dashed arrow)

In a 58-year-old right-handed woman diagnosed with an *IDH* wild-type left temporal glioblastoma, tb-fMRI was performed but was limited due to extensive patient motion; therefore, rs-fMRI was post-processed. A seed guided by ReHo was placed in left Broca’s area to obtain FC in left Wernicke’s area which was posterior to the glioma (Fig. 2).

#### Case 3: successful rs-fMRI after poor patient performance during tb-fMRI

**Fig. 3:**
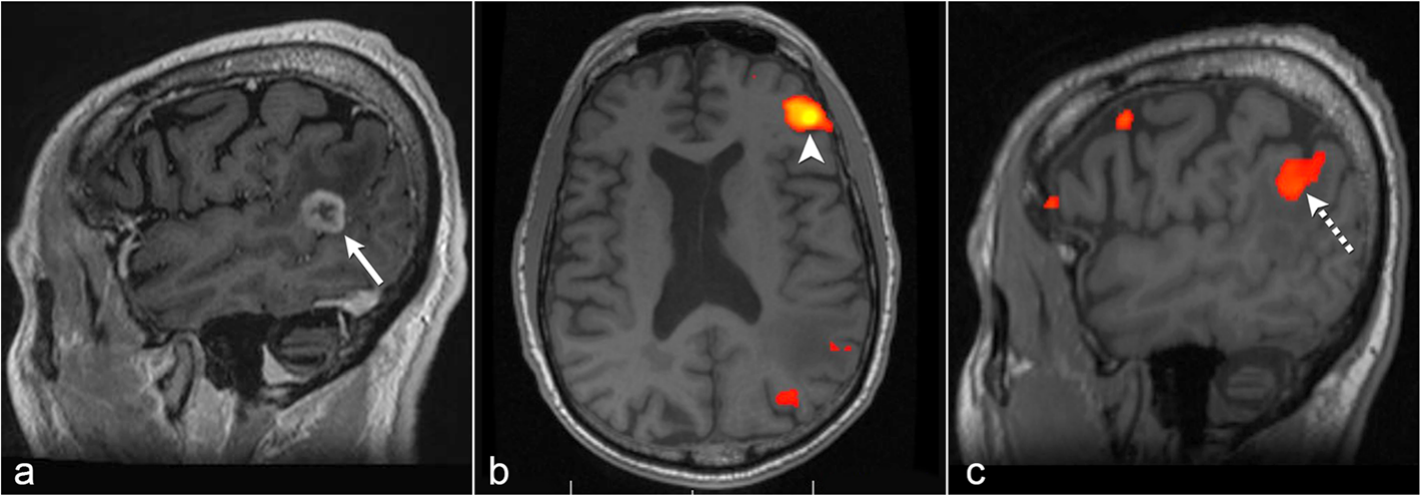
Successful resting-state fMRI after poor patient performance during task-based fMRI. Post-contrast sagittal T1 image (**a**) shows a left posterior temporal glioblastoma (arrow). Axial resting-state fMRI (**b**) with seed placement in left Broca’s area employing the regional homogeneity method (arrowhead). Sagittal resting-state fMRI (**c**) shows functional connectivity in the left inferior parietal lobule (dashed arrow) superior to the tumor

In a 73-year-old left-handed man with an *IDH* wild-type glioblastoma in the left posterior superior temporal gyrus, the patient could not follow instructions during tb-fMRI and therefore rs-fMRI was post-processed. Regional homogeneity was used to guide seed placement in left Broca’s area which demonstrated FC to the left inferior parietal lobule (Geschwind’s area) above the glioma (Fig. 3).

#### Case 4: successful rs-fMRI with seed placement in the contralateral hemisphere

**Fig. 4:**
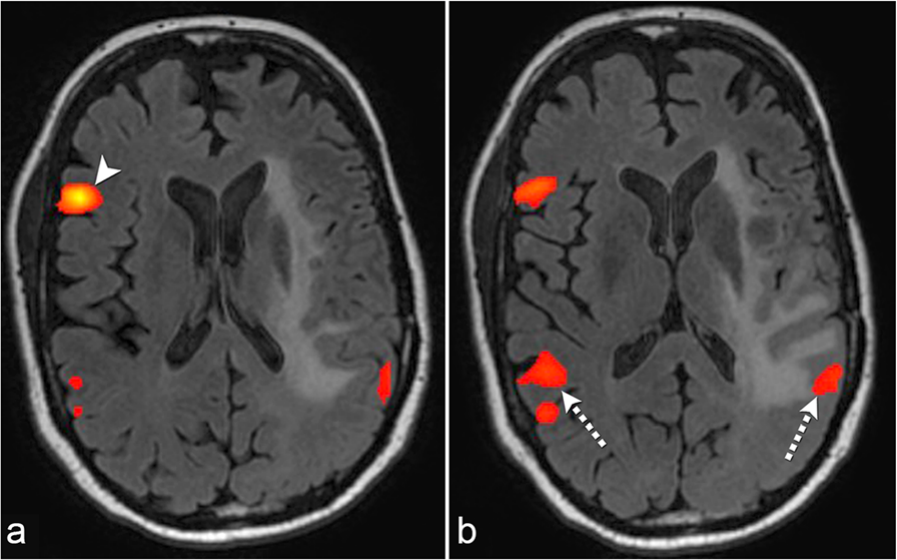
Successful resting-state fMRI with seed placement in the contralateral hemisphere. Resting-state fMRI (**a**) with seed in the contralateral right Broca’s area (arrowhead) using the regional homogeneity method in a patient with an infiltrating left insular glioma. Resting-state fMRI (**b**) shows functional connectivity in the bilateral Wernicke’s areas (dashed arrows)

In a 52-year-old right-handed man with an *IDH* mutant left insular grade II astrocytoma, tb-fMRI could not be performed due to patient impairment by tumor; therefore, rs-fMRI was post-processed. Initial seed placement in the left anterior language area did not elicit FC to the left posterior language area. Regional homogeneity was used to guide seed placement in the contralateral right posterior inferior frontal lobe, which demonstrated symmetric FC in the bilateral Wernicke’s areas. In the left Wernicke’s area, FC was noted abutting the FLAIR abnormality (Fig. 4).

#### Case 5: unsuccessful rs-fMRI due to patient motion

**Fig. 5:**
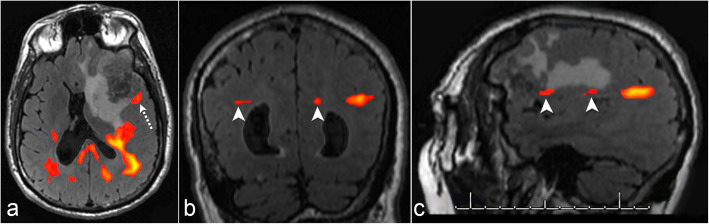
Unsuccessful resting-state fMRI due to patient motion. Axial resting-state fMRI (**a**) demonstrates possible functional connectivity (dashed arrow) posterior lateral to a left frontal brain metastasis. Coronal (**b**) and sagittal (**c**) resting-state fMRI shows functional connectivity only in a single plane (arrowheads) due to patient motion. This was deemed a resting-state fMRI failure

In a 62-year-old right-handed man with colorectal cancer who had a left frontal brain metastasis, tb-fMRI was attempted but was limited due to weak BOLD activation in the ALA; therefore, rs-fMRI was post-processed. A seed was placed at the tb-fMRI BOLD activation in the left PLA. rs-fMRI demonstrated potential FC posterolateral to the glioma. However, review of the sagittal and coronal rs-fMRI showed that FC was visualized only in a single plane, and therefore the rs-fMRI was deemed non-diagnostic. The single slice FC artifact was due to patient motion during the rs-fMRI acquisition. Patient motion can affect global FC (Fig. 5).

## Discussion

We evaluated the usefulness of resting-state fMRI for presurgical language mapping when task-based fMRI was limited in 49 patients with brain tumors. A seed-based correlation (SBC) approach used either tb-fMRI language activations apart from tumor or a regional homogeneity (ReHo) map to obtain functional connectivity (FC) to language areas of interest. Two neuroradiologists independently found the post-processed rs-fMRI data using SBC analysis was beneficial for functional language mapping in 41 (84%) and 43 (88%) cases respectively; Cohen’s kappa is 0.83, with a 95% confidence interval (0.61, 1.00) when tb-fMRI was limited. A subsequent consensus review by the two neuroradiologists found a total of 43 cases beneficial for language mapping. We found a 12% rs-fMRI failure rate for language mapping which was somewhat similar to a study using a multilayer perceptron rs-fMRI analysis which showed a rs-fMRI failure rate of 13% for combined motor and language mapping [15].

Further, six neurosurgeons found that the rs-fMRI results were “definitely useful” (26 cases; 60%) and “somewhat useful” (13 cases: 30%) in locating the ALA or PLA of clinical interest near tumor in their own cases. For example, one neurosurgeon commented in the questionnaires that the rs-fMRI results are “useful” in patients that are too impaired by tumor to perform substantive intraoperative direct cortical stimulation and have no tb-fMRI results. The rs-fMRI results serves as the only method of language brain mapping and could highlight a potential location to avoid (if possible) or approach with more caution during tumor resection.

In patients who performed poorly on speech paradigms during tb-fMRI (*n* = 12) and who were too impaired to even attempt tb-fMRI (*n* = 7); the post processed rs-fMRI FC was deemed beneficial by both neuroradiologists in 89% of cases for language mapping. It is in this subset of patients, that implementation of clinical rs-fMRI would be especially valuable. In instances when rs-fMRI showed robust FC in the same location as weak tb-fMRI activation (6 of 9 cases), this concordance increased diagnostic confidence in the localization of the language center. Therefore, rs-fMRI can also be used to verify equivocal tb-fMRI results.

When FC was observed in only one slice (Case/Fig. 5); the rs-fMRI data was considered non-diagnostic in all 6 (12%) cases. This unique rs-fMRI artifact is due to an undesired instability (i.e. patient motion) which can affect global FC. We acquire the rs-fMRI as the first fMRI sequence prior to all task fMRI paradigms to mitigate the chances of patient head motion. In addition, it is important to acquire the rs-fMRI sequence first to avoid the potential influence of cognitive tasks on the resting state networks.

Methods used to identify resting-state networks are classified into data-driven and hypothesis-driven methods. Resting state-ICA is a popular data-driven method which can display multiple networks simultaneously; however, it may not display the network of clinical interest. Further in practice, the network identified by ICA, if present, must be individually selected out by the investigator [6]. Resting-state SBC analysis is a hypothesis-driven method and more intuitive in targeting a specific language functional area of interest; however, a priori target must be determined, and this varies between studies [16]. In 19 patients, Cochereau et al. showed a significant correlation between language networks identified with SBC rs-fMRI and eloquent language cortex confirmed with direct cortical stimulation [17].

In 67% of the patients for whom SBC rs-fMRI was used in our study, tb-fMRI activation in the primary language area apart from tumor was used to guide seed placement. In the other 33% of patients, we used the ReHo method [18] to assist in seed placement for SBC rs-fMRI analysis. We did not have any cases in which the tumor or tumor mass effect distorted the anatomy to the degree that it affected rs-fMRI seed placement using tb-fMRI activations or ReHo. Anatomic landmarks alone were not used for seed placement in any of the cases due to known issues related to anatomic distortion caused by tumor or tumor mass effect. In a recently published research study performed at our institution, we compared SBC rs-fMRI maps generated using tb-fMRI activations (apart from tumor) as seed points, an automated ReHo method, and a canonical (anatomical landmark) approach. The ReHo method yielded rs-fMRI language mapping results that were in greater agreement with the results of tb-fMRI, with significant higher Dice coefficients (*p* < 0.5) than that of tb-fMRI and canonical approaches within the putative language areas [19]. Based on the results of this study, we plan to formally integrate an automated ReHo method to guide SBC rs-fMRI seed placement into our clinical workflow in the near future.

In 40 patients, FC was obtained in the hemisphere ipsilateral to tumor. Interestingly, in 3 patients in which FC could not be obtained from ipsilateral seed placement, the ReHo method was used to guide seed placement in the contralateral ALA to obtain successful FC to the ipsilateral to tumor ALA (2 cases) and ipsilateral to tumor PLA (1 case). Therefore, we have also shown in this study that contralateral ALA or PLA seeding is a potential viable secondary method of obtaining FC to the ipsilateral primary language areas of interest near tumor.

### Limitations

The number of limited tb-fMRI may be considered higher than expected due to inclusion of 6 weak tb-fMRI cases which were found to directly correlate with rs-fMRI FC results. Comparing tb-fMRI and rs-fMRI failure rate was not the aim of this study. The rs-fMRI was the first sequence obtained and we employed a closed eye rather than open eye technique to reduce potential eye strain for the subsequent tb-fMRI paradigms. We do not feel the patient falling asleep during the rs-fMRI acquisition is a confounding factor due to the use of a closed eye technique. In fact, there was no patient that had fallen asleep prior to the start of the tb-fMRI paradigms.

We did not investigate the potential reorganization of language function to the contralateral hemisphere that may occur in rare cases. Our goal was to identify a potential language area near the tumor using rs-fMRI. Further studies are needed to determine if rs-fMRI can reliably detect contralateral hemisphere language reorganization. We found seeding either the ALA or PLA resulted in co-activation of both ipsilateral and contralateral primary language networks in most cases. This coincides with findings from Doucet et al. [20], who found that SBC rs-fMRI identified broader and more bilateral language regions than did tb-fMRI. Therefore, at this time, we cannot determine hemispheric dominance with our current SBC rs-fMRI technique. We found a study by Lou et al. [21] which demonstrated that seed placement over the bilateral pre-supplementary motor area (pre-SMA) allowed identification of left language lateralization in 30 right-handed healthy patients. We did not use the bilateral pre-SMA as a seed and may utilize this area for future seed placement. Even without knowledge of hemispheric dominance, SBC rs-fMRI in this study could locate potential ALA and PLA functional language areas ipsilateral to tumor.

While the neurosurgical assessment compliments the neuroradiology assessment regarding the value of the SBC rs-fMRI technique used in this retrospective study; direct cortical stimulation data, percentage of tumor resection, complication rates including postoperative language deficits, and other clinical outcome data would be more informative. Lemée et al. found ICA rs-fMRI was able to detect language areas which correlated with cortical mapping with a sensitivity of 100%, compared to 65.6% with tb-fMRI [22]. We are currently undertaking a similar prospective analysis comparing SBC rs-fMRI, tb-fMRI, and cortical mapping at our institution.

## Conclusions

This study serves as a proof of concept that SBC rs-fMRI can be valuable for clinical preoperative language mapping when a patient cannot perform tb-fMRI or if the results are limited.

## Data Availability

No additional data.

## Abbreviations

ALA: Anterior language area
BOLD: Blood oxygenation level-dependent
FC: Functional connectivity
ICA: Independent component analysis
PLA: Posterior language area
ReHo: Regional homogeneity
rs-fMRI: Resting-state fMRI
SBC: Seed-based correlation
tb-fMRI: Task-based fMRI
